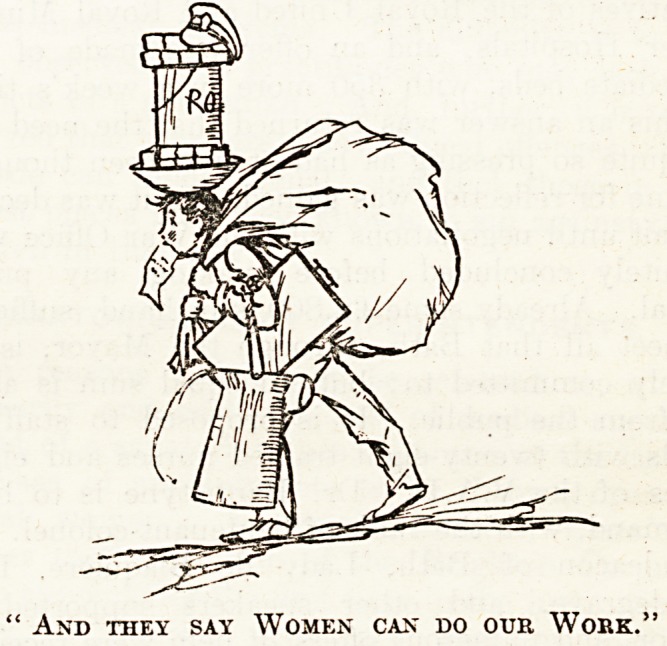# Hospital and Institutional News

**Published:** 1915-11-13

**Authors:** 


### HOSPITAL AND INSTITUTIONAL NEWS.

### ST. GEORGE'S HOSPITAL AS PIONEER.

The revised laws for the government of this hospital, which included many important alterations and changes, came into force on May 1 last. The alterations were material in the sense that they placed the management upon a new and business-like footing, and they enabled the reconstituted house committee to render active and material service to the efficiency of the hospital. We welcome evidence which is just to hand that the present administrative policy at St. George's is well up to date and worthy of an old and useful voluntary hospital. The voluntary hospital, as originally established, was for the benefit of those members of the civil population who, though able to maintain themselves and their families when well, could not when ill provide the cost of medical care and treatment. The laws of St. George's Hospital have always empowered the house committee to make grants out of the Samaritan Fund for the relief of the wives and families of patients during their residence in the hospital. Such grants are subject to the investigation and report of the almoner and the recommendation of the chaplain. It is now proposed to extend this power so as to enable similar grants to be made "during the patient's treatment or convalescence elsewhere under the orders of a medical officer of the hospital." No more beneficent or helpful change could be made, for it should preserve the self-respect and independence of the patient and his family whilst it keeps them self-respecting and free from the Poor Law.

### WORTHY OF UNIVERSAL ADOPTION.

In congratulating the new house committee of St. George's Hospital upon the beneficent addition they propose to law 78 (Samaritan Fund), we commend the adoption of a similar regulation, if it is not already in existence, to the authorities of every voluntary hospital for their prompt enforcement. This regulation maintains and strengthens the sound principle upon which our voluntary hospitals were originally founded. It will further commend St. George's Hospital, and every hospital that adopts the practice, to the generous and increased support of all who are interested in the adequate treatment and protection of the independent sick poor throughout the country.

### BRITISH WORKMEN OR MENTAL DEMENTS?

The Lord Mayor of Birmingham, Alderman W. H. Bowater, in distributing the cheques to the representatives of various hospitals and nursing societies on behalf of the Hospital Saturday Fund, made a most interesting speech. He pointed out that the workers throughout the city had risen to the occasion and come to the conclusion that Hospital Saturday must not suffer by the war. In the present year more than ever was the help of the citizens needed by the hospitals, and the results of the collections justified the expectation that the sum raised would this year exceed £23,000. The Lord Mayor then proceeded to state that the new Children's Hospital at Ladywood, planned to accommodate 216 children, had for nearly twelve months been left in a state of incompletion because "the plumbers engaged went on strike for the reason that they could not agree with the hot-water engineers as to who should finish the hot-water fittings." In fact, a new hospital had been prepared, the site secured, the foundation-stone laid by Royalty, a sum of £30,000 collected, and the work of erection proceeded with almost to completion when the plumbers and hot-water engineers stopped the works. They had for many months deprived the people of Birmingham of the comfort and protection of a magnificent hospital, which would have provided for the treatment of hundreds of sick children belonging to the workers. To make the matter worse, the action of these artisans had prevented the old Children's Hospital buildings being available for the treatment of sick and wounded soldiers, for whom accommodation had to be provided at a further loss to the children and their parents from the closing of three elementary schools and an expenditure of thousands of pounds to turn the latter into hospitals for the soldiers. On behalf of the citizens might not the medical profession of Birmingham meet and appoint five of the ablest men amongst them to hold an inquiry into the sanity of the plumbers and hot-water engineers in question? They might so find it possible to protect the citizens from further follies and dangers of the kind. No such inhuman folly would have been tolerated in the days of Joseph Chamberlain, for Birmingham men in his day were famed for their intelligence, their citizenship, and their power and will to deal with cranks by cutting their cackle.

### CIVIL PATIENTS IN WAR TIME.

It may be true that one effect of the war has been to make a liberal percentage of the population, formerly often in the doctor's hands, so robustly well as not now to need their services. In the case of one London hospital, where the waiting list was large and each day's out-patients provided twice as many in-patients as there were vacant beds, the position is now reversed. Not only has the number of civil in-patients seeking admission fallen considerably, but room has been readily found for a large number of wounded cases, and there are always vacant beds for civil cases. At Manchester, where before the war the Royal Infirmary authorities opened two central branches for "out" and accident cases, one of these had to be closed owing to a shortage in the medical staff. The other, the Roby Street (Piccadilly) branch, not only nurses military patients, but does accident work. The closing of the Parker Street branch caused many accident cases to be sent from the centre of the city to the new infirmary, involving a delay of some twenty minutes, which Mr. Carnt, the superintendent, intimates may be obviated by taking them direct to Roby Street. We have here exhibited one effect of the war on civil cases. Mr. Carnt's letter demonstrates the desire of voluntary hospital authorities to minimise all such difficulties as much as possible.

### A NEW YORKSHIRE SANATORIUM.

An interesting new institution, the Middleton-in-Wharfedale Sanatorium, has just been opened by Mr. T. Benson P. Ford, chairman of the West Riding Public Health and Housing Committee. The site, which is extensive, comprises 145½ acres, and the institution is placed on one of the plateaus which are a feature of the ground. This natural advantage lends readily to the adoption of the pavilion system, and at present two pavilions have been completed, each of which accommodates twenty-five patients, men and women respectively. Besides these two blocks fifty more men and women are accommodated in shelters, so that the institution opens with accommodation for 100 patients. If the plan be completed two more blocks, each for 100 patients, will be added, and eventually, it is hoped, that a total of 300 patients may be installed. The cost per bed of the 100 beds now in use, including, we understand, land, building, and equipment, amounts to £170; the cost per bed of the second hundred should be £162, and the third, it is hoped, will be £136. Surely the growth of sanatoria, in spite of reactions against the boom which they received at the hands of politicians some years ago, is only another proof that through good and through evil report the sanatorium treatment of tuberculosis is the only one which has survived and persisted.

### THE VALUE OF THE PRIVATE STAFF.

Those hospitals with private nursing staffs have had to suffer a loss in their accounts, as is apparent from those lately discussed at a meeting of the Devon and Exeter Hospital. The receipts from the fees for private nursing have fallen off by 50 per cent., owing to the nurses having left to take up war work. This has meant a loss of £328 for the past quarter, against which can only be set the cost of maintaining the staff. On the other hand, the public has continued to support the institution, and the fact that the hon. treasurer has been able to congratulate the governors on this fact once more points out the old truth that a well organised private staff can be of great value to a hospital in maintaining its reputation and popularity among the general public.

### NO DRIVING FORCE AT WORCESTER INFIRMARY.

At a recent meeting of the executive committee of this institution, the Corporation of Worcester requested that they might be allowed to have the use of the infirmary mortuary. The mortuary was described by a Mr. Harris as being "in a poor state, but a present of a mausoleum resembling that at the Kidderminster Infirmary would be very acceptable." The state of affairs at Worcester, hygienically, may be fairly surmised from the fact that the Dean is reported to have thought that such an arrangement might be possible, for otherwise the city would have to build a mortuary! The Chairman, whose name is not given, strongly and properly disapproved of any arrangement being made with the Worcester Corporation which would involve a dual control of the mortuary. Apparently the fact is the Worcester Infirmary is not at present the possessor of a mortuary in any modern meaning of the word. Such a fact would reflect little credit on the administration and management of this institution. It is not satisfactory that the Corporation's request should have been left for further consideration instead of being refused point blank, for it would seem to have been opposed to the best interests of the infirmary and of the inhabitants of the city of Worcester. The internal condition of affairs indicated by the discussion on the mortuary may possibly have something to do with the fact that the infirmary is still without a resident medical officer, although several applications for this post have been received. For the report is that invariably the committee were so dilatory in coming to a decision that in every case the applicant was snapped up by some other institution.

### BEAUFORT WAR HOSPITAL AND THE BRISTOL GUARDIANS.

The Bristol Guardians have been requested by Colonel Blachford, of the Beaufort War Hospital, to provide accommodation for 115 sisters, probationers, and servants, the building previously offered by the Board being sufficient for about eighty. Colonel Blachford asked for two more wards to complete the necessary accommodation. The Guardians declined to accede to this request, on the grounds that they had already handed over the female infirmary block promptly on being asked for it, and that to give up two wards in the male block would cause too great an inconvenience to make it possible. The arrangements for shifting the female patients had caused considerable trouble, but were now completed, and the Guardians, apparently, refused to begin all over again. Certainly there are limits, though whether the Bristol Guardians were right to decline the request made to them only those with full knowledge can decide.

### AN AGE LIMIT AT RADCLIFFE INFIRMARY.

At the last quarterly court of the governors of the Radcliffe Infirmary and County Hospital, Oxford, the energetic Chairman (the Rev. G. B. Cronshaw) proposed that henceforth there shall be an age limit of sixty-five for the physicians and of sixty for the surgeons of the visiting staff. This regulation was adopted by the meeting, and is to apply to the last appointed surgeon (Mr. Bevers) and to all future appointments. The older members of the staff, or most of them, may voluntarily place themselves within this new rule. It is found to be beneficial at the numerous hospitals which have adopted it, and we should like to see it become general all over the country. Other matters of interest before the court were, first, a resolution giving the committee power to raise the salaries of the resident medical officers from £80 to £200 (or less); and, second, some facts about the war work being done, from which it appears that thirty-seven soldier patients were treated, many of them for long periods, by electrical methods. Further, the Radcliffe Infirmary is doing the electrical work for the Base Hospital, the operations at the officers' hospital at Somerville, and a great deal of sterilising as well. Clearly "the Radcliffe," like other voluntary hospitals, has risen to the occasion; and it is gratifying to note that extra support has been forthcoming to meet the extra expenditure thus incurred.

### WAR FUNDS AT LIVERPOOL.

In cold print the sums raised for various relief and war funds in Liverpool look imposingly vast; but according to the *Liverpool Daily Post and Mercury* both Liverpool and Manchester have failed in their duty compared with Glasgow. The aggregate contributed at the Clydeside city was £882,382 some weeks ago, it is stated; whereas to the end of October Manchester's total was £271,065 and Liverpool's £264,166. The paper we have quoted blames want of organisation for the Liverpool results, and particularly the Lord Mayor and the civic authorities, who wet-blanket schemes organised or suggested by others, and are lethargic themselves. It is said that no proper attempt to enlist and maintain the interest and support of the artisan classes has been made; and that Flag Days and tram-car boxes have been discouraged instead of welcomed. A wealth of statistics is displayed in support of these contentions; and certainly, if Glasgow can produce the vast sums contributed to all sorts of war charities, Liverpool and Manchester are well able to do more than they have done so far. On the facts the *Liverpool Daily Post and Mercury* has made out a good case calling for answer and investigation.

### ANOTHER BRITISH HOSPITAL IN THE ARGONNE.

Not long ago we noted Mr. Paget's appeal for the Serious Cases Hospital, which ministers to the French wounded in the Argonne district. Now Mr. Laurence Binyon, the poet, pleads for support of another British hospital in the same region—that at Arc-en-Barois. It appears that an annexe hitherto used as a convalescent depôt has been added to the accommodation for serious cases, and this, of course, entails increased expenditure. There are 180 beds, and the estimated monthly expenditure is about £900. Mr. Binyon gives a touching picture of the gratitude of the French soldiers to their English doctors and staff; and there can be no doubt that international amity and understanding are greatly enhanced and distributed by this and similar works of mercy.

### THE STATE CHILD.

Circumstances are making mincemeat of the old controversy over the family *versus* the institution for the upbringing of those children whose homes are broken up through death, crime, or poverty. If it had not been for the war it is believed that all children would have been removed from the workhouses through the efforts of those who have made it their business to point out the evils of leaving young children in the necessarily mixed company of largely unclassified workhouse inmates. As things are, the completion of this essential removal has been postponed. But while the State child, meaning the child bred in a mixed State institution, is a much criticised product, the aid of the State is being more than ever invoked on behalf of those who have been the victims of the war. The French, we understand, are making special provision for war orphans, on the boarding-out principle, and it seems probable that the workhouse child will soon become a thing of the past, and that the organisation of boarded-out children will emerge from the somewhat haphazard conditions of what has been little more than an experiment into a regular department of institutional work.

### A REMARKABLE HOSPITAL MAGAZINE.

We have to thank the Editor of the *Gazette* of the 3rd London General Hospital, Wandsworth, for sending us his second number, which has many points of interest even to the outside world. Although most of the articles are naturally designed to have a personal interest for the hospital's staff and patients, there is a creditable attempt to preserve a high standard in the letterpress, and the result is something better than "a parish magazine." Beyond question the best feature in this

"And they say Women can do our Work."

issue is represented by the illustrations; but then it is not every hospital which numbers among its staff such an able draughtsman as Mr. C. R. W. Nevinson, whose black-and-white drawing entitled "Night Arrival of the Wounded" is a really delightful example of vivid illustration. Apart from its decorative quality, on the ground of vividness alone, his futurist method is justified. Very different in aim, but pleasing in its own fashion, is Private Paul Kirk's "Hospital by Moonlight." Both illustrations show that black-and-white work is the ideal for illustrative purposes, and puts photographs completely in the shade. We trust that the Editor will make the work of these two draughtsmen a regular feature. If he does his *Gazette* will have an assured place among hospital magazines. Private De la Bere's humorous sketches (particularly No. 7) are also good, and the editorial on the "Woman Orderly" is of general interest.

### MILITARY CONVALESCENTS AT BLACKPOOL.

A further three hundred beds have been added to the Lancashire Military Convalescent Hospital at Blackpool, and there are rumours of yet further expansion. A great deal of voluntary work is being done by local patriotism to make pleasant the lot of those admitted to this institution. A C.E.T.S. billiard-room has been opened, with four tables built by Riley of Accrington; there is an ante-room for chess and similar indoor games. The erection of an Empire Hut (Women's Volunteer Reserve canteen) has been embarked upon; and bands are playing twice a week for the patients' enjoyment. Special requirements not yet filled consist of bulbs and shrubs for the garden, books (not magazines) for the library, walking-sticks, old tennis-balls, and woollen gloves without fingers. Evidently Blackpool is alert for the comfort and well-being of its guests at this hospital.

### BATH'S PROPOSED WAR HOSPITAL.

At a largely attended public meeting in the Guildhall of Bath, the Mayor explained that in May last he received a telegram from the War Office urgently begging for a war hospital of 500 beds. A conference was at once held with the local branches of the British Red Cross Society and of the St. John Ambulance Association, and with representatives of the Royal United and Royal Mineral Water Hospitals, and an offer was made of 150 immediate beds, with 350 more in a week's time. To this an answer was returned that the need was not quite so pressing as had at first been thought; so time for reflection was gained, and it was decided to wait until negotiations with the War Office were definitely concluded before making any public appeal. Already some £2,800 is in hand, sufficient to meet all that Bath, through the Mayor, is definitely committed to; but an equal sum is asked for from the public. It is proposed to staff the wards with twenty-eight trained nurses and eighty ladies of the V.A.D. Dr. Bannatyne is to be in command, with the rank of lieutenant-colonel. The Archdeacon of Bath, Lady de Blaquière, Lady Waldegrave, and other speakers supported the Mayor, and numerous offers of help were received. Complete unanimity seems to have prevailed at the meeting, which filled the Banqueting Hall, and was evidently representative.

### COUNTESS TEMPLE'S PROTEST.

A dissentient note has been struck, however, by Countess Temple, in a letter to the local Press. This lady states that the proposed new hospital "in the opinion of many people is not wanted": this is a poor argument, since the War Office want it, and no one can dispute their knowledge of the country's needs. She says that the military hospitals of the Southern Division, as well as the V.A.D. hospitals, have been frequently half-empty. Also that Lord Temple and herself have spent in ten months over £1,000 for their hospital at Newton Park, without begging for help from the public. This, again, is a weak argument, for clearly Lord and Lady Temple cannot have had 500 beds open. The letter proceeds in acid tones, "the City of Bath being apparently delighted to defray the expenses of an unwanted luxury in the shape of the new hospital, we shall with great reluctance be compelled to close Newton Park Hospital, as evidently it will be no longer needed. I write this letter as a protest against this extravagant undertaking, and we certainly do not intend to subscribe to it in any way." No comment seems called for, except the hope and belief that Lady Temple has already regretted the wording and publication of her letter.

### MEDICAL ARRANGEMENTS IN MESOPOTAMIA.

While less inadequate attention is beginning to be paid to the campaign in the Persian Gulf, of which events in the Balkans are now illustrating the importance, it is disappointing to have so little information concerning the medical arrangements in this theatre. In reply to a recent question in the House, Mr. Austen Chamberlain declared that details on this subject were not available in this country, but declared that the general hospital at Basra was well equipped and that "other hospitals have been established at various points in the sphere of operations." Connection with India is maintained by hospital ships, and the Government of India is responsible for the taking of sufficient measures to ensure an adequate medical service. It is hardly necessary to remind our readers that the varying reports concerning the condition of the medical arrangements in Egypt some months ago supplied a keener edge to criticism from the paucity of authentic information concerning them. No doubt the more distant the campaign the more difficult it is to supply details, but in view of recent events it is important to know the scale on which the medical arrangements in the Persian Gulf have been organised. It will be remembered that both British and Indian troops are engaged in this quarter.

### THE NEW HOSPITAL IN OAKDALE MODEL VILLAGE.

The result of the Tredegar Company's effort to improve the housing conditions of their miners is the Oakdale model village, which is now in process of being built in Monmouthshire. One of the first public buildings to be opened is the cottage hospital, the foundation-stone of which was laid on July 13, 1914. Designed by Mr. A. E. Webb, the institution, which provides eleven beds, has been erected for £4,000, of which rather more than half has been raised, mainly by workmen's subscriptions, and partly by a gift from the Tredegar Company. The secretary of the hospital is Mr. Oliver Harris, who declared at the recent opening ceremony that royalty owners should be under a legal obligation to assist the institution. In this respect the company seem to have set an example, since they have provided the site at a nominal rent of £1 per annum and given a donation of £250. Since the workmen by subscribing a penny in the pound out of their wages have raised £2,300, it is only fair that the owners should combine to provide the remaining half of the money required to pay for the erection and equipment. The chairman of the hospital committee is Mr. D. Aggex.

### HOSPITAL WOOD-CARVING.

Notwithstanding the economic difficulties in the way of voluntary work in the manufacture of goods for the benefit of hospitals, such efforts continue to be made in ever-new directions. A good instance of a new departure is to be found in the recent scheme inaugurated by Mr. W. Mold at Pokesdown, in Hampshire. This gentleman, a member of the staff of the Bournemouth Municipal College, is in charge of the woodwork and carpentry classes. It occurred to him that classes might be organised to make articles required by the local hospitals. With the consent of the education authority, a workroom has been started and a number of persons with some skill in woodwork and joinery have offered their services. The classes are held from 6.30 to 9.30 every Wednesday evening at the Technical Hall, Pokesdown, and the articles in process of manufacture are destined for the Christchurch Hospital. Such articles as bed-trays, splints, and folding tables are "on order." It would be interesting to see some specimens of this work, and to hear how the scheme is regarded by the local tradesmen.

### A HOSPITAL DIAMOND JUBILEE.

The Poplar Hospital for Accidents was founded in 1855; at a time, that is, when the nation was allied with the French in war against the Russians. It has therefore attained this year its Diamond Jubilee, but owing to the war there will be no celebration festival. It is, however, proposed to hold a meeting on behalf of the hospital at The Baltic on Wednesday, December 15. Lord Inchcape will preside, and Lord Knutsford (chairman of the committee) and Mr. Crooks, M.P., will be among the speakers. The decision to hold no festival is probably wise; but we hope the chairman's well-known powers of persuasion will result in a substantial list of donations and subscriptions from The Baltic meeting.

### HOSPITAL PHYSICIAN CHANGES HIS NAME.

Dr. Otto Fritz Frankau Grunbaum, physician to the London Hospital, has written a letter to the *Lancet* to announce that he has changed his name to Leyton, and to explain that he wishes to dissociate himself as far as possible from a nation the deeds of which make it lose any right to be included amongst civilised races. Those who know Dr. Leyton will need no assurance as to his thoroughly British sympathies, but his attitude in desiring to cut himself loose from even the suspicion of Germanophil tendencies is quite natural and understandable. Dr. Grunbaum, as he then was, had a distinguished career at Cambridge, where he took first-classes in both parts of the Science Tripos. He was afterwards educated at St. George's, and is an instance—of which there are many in London—of a doctor educated at one hospital obtaining a staff appointment at another.

### A NEW SUBJECT FOR MEDICAL STUDENTS.

A proposal was brought forward by Dr. McVail at the recent session of the General Medical Council that the Education Committee should report on the education of medical students in the ethical relations of medical practitioners to the State, to their patients, and to each other. After some discussion the committee, with the addition of Drs. Newsholme and McVail, was empowered to inquire into the matter. The most important objection raised to the proposal was that expressed by Dr. Mackay, that the step suggested might encourage the mistaken view held by some members of the public who think that medical ethics is a system of rules akin to those of a trade union and designed to make as much money out of the public as possible. Sir Thomas Fraser's objection was that the atmosphere of the schools and of the profession generally is the best corrective of aberrations in medical ethics. We agree that these atmospheres are wholesome, but the difficulty is to influence thereby a certain type of practitioner who is wholly alienated from them. In the same way we rather distrust educational methods, which mean presumably lectures, text-books, and examinations. Medical ethics, so mysterious to the public, are based on the ordinary canons of gentlemanly behaviour applied to certain special medical problems. The difficulty is to get them observed by a practitioner who may lack the instincts of a gentleman. And, further, it is to be observed that there are many ethical offences which no code can render penal; and that those in the highest places have been known to sin against their brethren in this way.

### SANATORIUM LADY SUPERINTENDENTS.

For reasons which may be guessed at, if not necessarily endorsed, the post of medical superintendent of a sanatorium has been one of the last of this class of appointments to be thrown open to medical women. A certain interest, therefore, attaches to the decision of the Brighton Corporation to appoint, for the first time in the institution's history, a woman temporary resident medical officer to the Borough Sanatorium. The previous medical officer, Dr. Adam, having joined the Forces, the Corporation has appointed Mrs. Eveline Rosetta Cohen as his successor. This lady, who graduated M.B., Ch.B. at Edinburgh in 1909, and is an F.R.C.S. of Ireland, is, it is interesting to note, a Colonial by birth, having been born in Sydney, the daughter of Mr. Samuel Benjamin, of Hobart, Tasmania. Her previous appointments include that of resident medical officer at the Victoria Jewish Hospital, Manchester, and she has lately acted as a V.A.D. commandant in London. Mrs. Cohen has been appointed, for at least one year, at a salary of £250.

### THE NORFOLK SUPPLY DEPOTS.

The movement for the creation and establishment of war hospital supply depôts, notwithstanding the economic difficulties liable to occur when voluntary workers start to make goods from the manufacture of which certain classes gain their livelihood, continues to grow. Many county organisations are in existence, and there is a certain amount of affiliation to London. Norfolk provides a good example of the county system. There is a central depôt at Norwich, and others at Cromer and elsewhere in the county, from which goods are dispatched not to home hospitals, but for use abroad. The central depôt consists of four rooms—the needlework, bandages, surgical, and slipper rooms—and there is a staff of eighty-six workers. The way to become a worker is by the payment of a fee which ranges from threepence to half a crown per week. The sum thus collected is apparently devoted to the purchase of materials. The depôt is open twice a week from ten to three, and light refreshments can be obtained at nominal cost on the premises. Each room has its superintendent, and the remainder of the staff comprises a treasurer, an accountant, and the honorary secretary, Mrs. A. Collison. It is through the central depôt that goods are distributed abroad.

### THE DOCTOR'S WIFE AS "LOCUM."

It is not often that a medical man holding an official appointment is able to secure his own wife as *locum tenens* during his absence, but the medical officer of health for the county of West Sussex is in this fortunate position. He has accepted a temporary commission in the R.A.M.C., and the approval of the Local Government Board has been obtained in respect of the appointment of Mrs. F. E. Smedley to the post of acting county medical officer, school medical officer, and medical adviser to the West Sussex Insurance Committee, the last in place of the tuberculosis officer, who is absent in France. Mrs. Smedley qualified from the London School of Medicine for Women as M.B., B.S. (London) in 1905. Both she and her husband have held appointments in Sheffield.

### SCHOOL CLOSURE AGAIN.

At this time of the year there is nearly always a rising tendency in the incidence of zymotic diseases, and the perennial question of school closure is sure to crop up. The question is one which cannot be answered in a few words, but this may be said without fear of contradiction: the expediency of closing schools except under most unusual circumstances is steadily becoming regarded with more and more disfavour. A watchful teacher is a great asset and a good safeguard; the emptying of children into the streets and courts of towns is an excellent way to propagate an epidemic. In the case of measles it is obviously totally unnecessary to forbid the school to those children who have already had this illness, and a measles register is now to be found in every well-conducted school.

### PORTUGUESE AID FOR BRITISH WOUNDED.

A project has been set on foot in Portugal for arranging hospital accommodation for British wounded from the Dardanelles and from Serbia. Seven Portuguese watering-places have been selected as suitable localities for the reception of our casualties; and the Government has, it is said, offered to make structural alterations of any buildings offered by private generosity in order to fit them for hospital purposes. Hotels, sanatoria, and hospitals are being inspected in order to determine their suitability for such conversion. In addition to this, it has been proposed in the Portuguese Parliament that all the public sanatoria and grounds in the island of Madeira be handed over to the British Red Cross Society for the duration of the war; and even that there shall be a suspension of import duties on all necessary stores and supplies. These very generous proposals serve to emphasise the traditional amity between the Portuguese nation and ourselves. We have, too, recently been celebrating the centenary anniversaries of so many of the battles in which we were jointly engaged to defeat an attempt to impose a military tyranny upon Europe almost as disastrous as that which the German Emperor is attempting to-day.

### THE CINEMA AMBULANCE FUND.

On November 5 Sir William Treloar presided at a luncheon at the Trocadero given by anonymous supporters of the Cinematograph Trade Ambulance Fund. It was reported by the Chairman of the London Exhibitors Association that the fund was being well supported in the provinces, but that only 160 out of 378 proprietors in London had pledged themselves to hand over their profits of Nov. 9 to the fund. During the afternoon various donations were received, including one of £500. An auction sale of tickets for the Opera House *matinée* of November 16 for the same fund was also held: three stalls fetched 30 guineas, and a private box brought in 45 guineas; in all £225 was raised. The scheme is evidently being well received and organised, though it is distinctly disappointing that so many cinematograph proprietors in London refuse to aid it.

### THIS WEEK'S DRUG MARKET.

The general advancing tendency in the prices of drugs continues, although there are a few exceptions to this rule. For instance, quinine has rapidly diminished in price as a result of the Order in Council prohibiting its export; this step might with advantage have been taken much earlier, and it certainly does not appear to be sound policy to allow our stocks of a much-needed drug to be depleted to an unnecessary extent. Epsom salt is obtainable at lower rates, in consequence of supplies being more plentiful. In sympathy with the continued and increased scarcity of belladonna, atropine is again dearer. Emetine has advanced in price as a result of the high value of ipecacuanha. Bromides are again dearer, and extraordinary prices have been paid for ammonium bromide. The high quotations for synthetic drugs are well maintained. Menthol has again advanced in price. Senna leaves are now more plentiful, and prices are slightly lower.

## Figures and Tables

**Figure f1:**